# Gene Expression Signature of Traumatic Brain Injury

**DOI:** 10.3389/fgene.2021.646436

**Published:** 2021-03-30

**Authors:** Yawen Ma, Yunhui Liu, Xuelei Ruan, Xiaobai Liu, Jian Zheng, Hao Teng, Lianqi Shao, Chunqing Yang, Di Wang, Yixue Xue

**Affiliations:** ^1^Department of Neurobiology, School of Life Sciences, China Medical University, Shenyang, China; ^2^Department of Neurosurgery, Shengjing Hospital, China Medical University, Shenyang, China; ^3^Key Laboratory of Neuro-Oncology in Liaoning Province, Shenyang, China

**Keywords:** traumatic brain injury, biomarkers, neuroimmunology, bioinformatics, signature genes

## Abstract

**Background:** Traumatic brain injury (TBI) is a brain function change caused by external forces, which is one of the main causes of death and disability worldwide. The aim of this study was to identify early diagnostic markers and potential therapeutic targets for TBI.

**Methods:** Differences between TBI and controls in GSE89866 and GSE104687 were analyzed. The two groups of differentially expressed genes (DEGs) were combined for coexpression analysis, and the modules of interest were performed using enrichment analysis. Hub genes were identified by calculating area under curve (AUC) values of module genes, PPI network analysis, and functional similarity. Finally, the difference in immune cell infiltration between TBI and control was calculated by ssGSEA.

**Results:** A total of 4,817 DEGs were identified in GSE89866 and 1,329 DEGs in GSE104687. They were clustered into nine modules. The genes of modules 1, 4, and 7 had the most crosstalk and were identified as important modules. Enrichment analysis revealed that they were mainly associated with neurodevelopment and immune inflammation. In the PPI network constructed by genes with top 50 AUC values in module genes, we identified the top 10 genes with the greatest connectivity. Among them, down-regulated RPL27, RPS4X, RPL23A, RPS15A, and RPL7A had similar functions and were identified as hub genes. In addition, DC and Tem were significantly up-regulated and down-regulated between TBI and control, respectively.

**Conclusion:** We found that hub genes may have a diagnostic role for TBI. Molecular dysregulation mechanisms of TBI are associated with neurological and immune inflammation. These results may provide new ideas for the diagnosis and treatment of TBI.

## Introduction

Traumatic brain injury (TBI) is an important public health problem as it is one of the leading causes of death and disability in the world (Hyder et al., 2007). Globally, more than 50 million people suffer from TBIs every year (Maas et al., 2017). The pooled annual incidence for mild, moderate, and severe TBI are 224, 23, and 13 per 100,000, respectively (Nguyen et al., 2016). The peak incidence of TBI occurs in youth and older life, and it will cause morbidity and mortality in young people under 45 years of age. The huge expenditure on clinical management of TBI patients and related socio-economic problems have brought a heavy burden to the medical system and society (Peters and Gardner, 2018).

Explosion and impact are the main causes of TBI, which lead to a certain degree of cerebrovascular injury, white and gray matter damage, and neuronal and/or glial cell damage (Rodriguez et al., 2018). TBI has now been associated with post-traumatic stress disorder, memory deficit, chronic traumatic encephalopathy (CTE), and chronic neuroinflammation (Goldstein et al., 2012). In addition, TBI also increases the risk of additional health problems for individuals, such as depression, neurodegenerative diseases, and post-traumatic epilepsy (Bolton-Hall et al., 2019). TBI is a diverse process that involves the interaction of many pathophysiological events and processes (Povlishock and Katz, 2005). This poses a major challenge in identifying reliable and sensitive biomarkers in TBI. At present, no TBI biomarker has been found that can be reliably used for clinical diagnosis and prognosis.

Mitochondrial dysfunction is one of the characteristic events of TBI (Xiong et al., 1997). Increasing evidence suggests that oxidative stress plays an important role in the pathogenesis of TBI (Ansari et al., 2008). In TBI patients, the sustained up-regulation of various inflammatory factors is associated with changes in permeability, edema formation, and neurological deficits during the process of blood–brain barrier damage (Ng and Lee, 2019). The role of the immune system in the pathogenesis of TBI has attracted increasing attention. It has been suggested that immune regulation may significantly alter the clinical outcomes of TBI patients (Jassam et al., 2017).

Bioinformatics analysis tools can both identify key molecules and elucidate their interactions. This study explores potential biomarkers and therapeutic targets through TBI-related gene expression in public databases. Further understanding of specific pathophysiological mechanisms leads to TBI-related dysfunction. These results provided opportunities for preclinical and clinical research to improve our understanding of the pathogenesis of TBI and promoted the development of effective treatments.

## Materials and Methods

### Data Sources

We collected TBI-related data from the gene expression omnibus (GEO) database. The gene expression profiling processed on GPL16791 of GSE89866 included blood samples from 29 individuals at baseline and after experiencing a moderate blast exposure, respectively. GSE104687 included gene expression profiling of brain samples from 93 TBI to 103 no TBI individuals without loss of consciousness processed on GPL16791. Cortical gray (parietal and temporal) and white matter (parietal) and hippocampus samples were included. FPKM data matrix was first adjusted for the total transcript count using TbT normalization and then log-transformed.

### Analysis of Differentially Expressed Genes

The DEGs were obtained from TBI and control subjects through limma R software package (Ritchie et al., 2015). The *P*-value < 0.05 was the threshold for nominally significant differential expression.

### Weighted Correlation Network Analysis

The coexpression network analysis was performed on TBI and control samples using WGCNA R software package (Langfelder and Horvath, 2008). Selected a power of β value and set the minimum module size as per the standard scale-free networks. Following eigengene calculation, correlation of eigengenes was identified by WGCNA to the clinical traits.

### Enrichment Analysis

The enrichment analysis of gene ontology (GO) functional analysis and Kyoto Encyclopedia of Genes and Genomes (KEGG) pathway analysis was performed for important module genes through clusterProfiler R software package (Yu et al., 2012; Gu et al., 2020a,b). The results of gene enrichment were quantified using gene set variation analysis (GSVA) R package. GSVA scores were calculated using a Kolmogorov–Smirnoff-like random walk statistic and a negative value for a particular sample and gene set. Gene Set Enrichment Analysis (GSEA) of genes in TBI and control was carried out using GSEA software. The *P*-value < 0.05 was considered statistically significant.

### Protein–Protein Interaction Network

The protein–protein interaction (PPI) network was constructed by putting selected genes into Search Tool for the Retrieval of Interacting Genes (STRING) (https://string-db.org) (Shi et al., 2018a,b). Hub genes were obtained through degrees of connections with other genes in PPI network. PPI network is displayed through Cytoscape.

### Infiltration of Immune Cells

The marker gene set for immune cell types was obtained from Bindea et al. (2013). Single-Sample Gene Set Enrichment Analysis (ssGSEA) program was used to quantify the infiltration levels of immune cell types. The ssGSEA applies gene signatures expressed by immune cell populations to individual samples.

## Results

### Coexpression Network of DEGs

**Figure 1:**
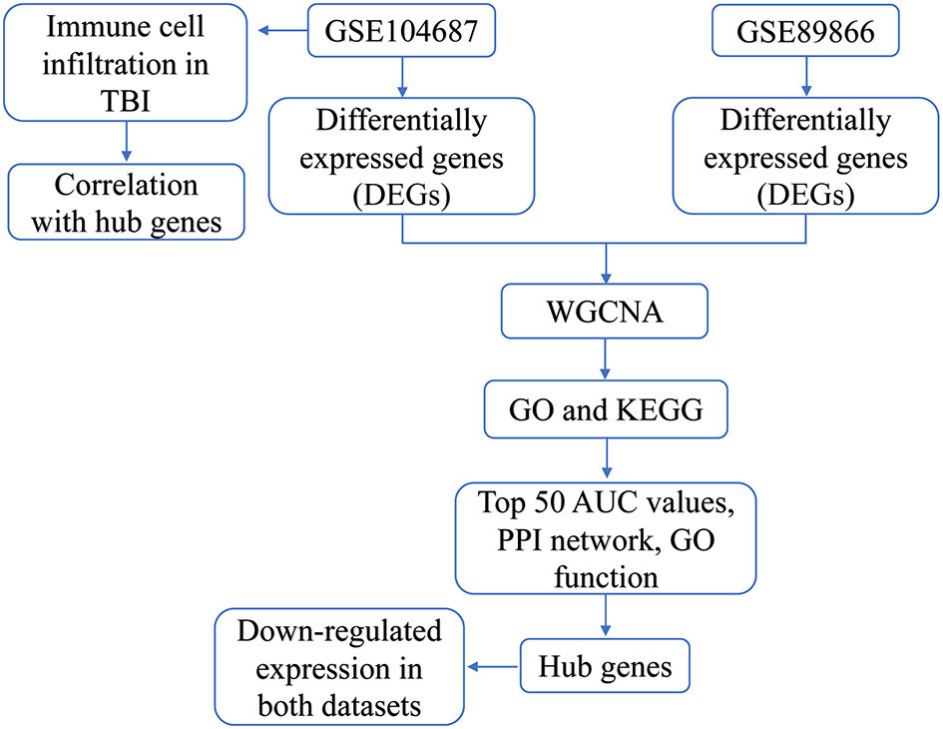
The flowchart of this study.

**Figure 2:**
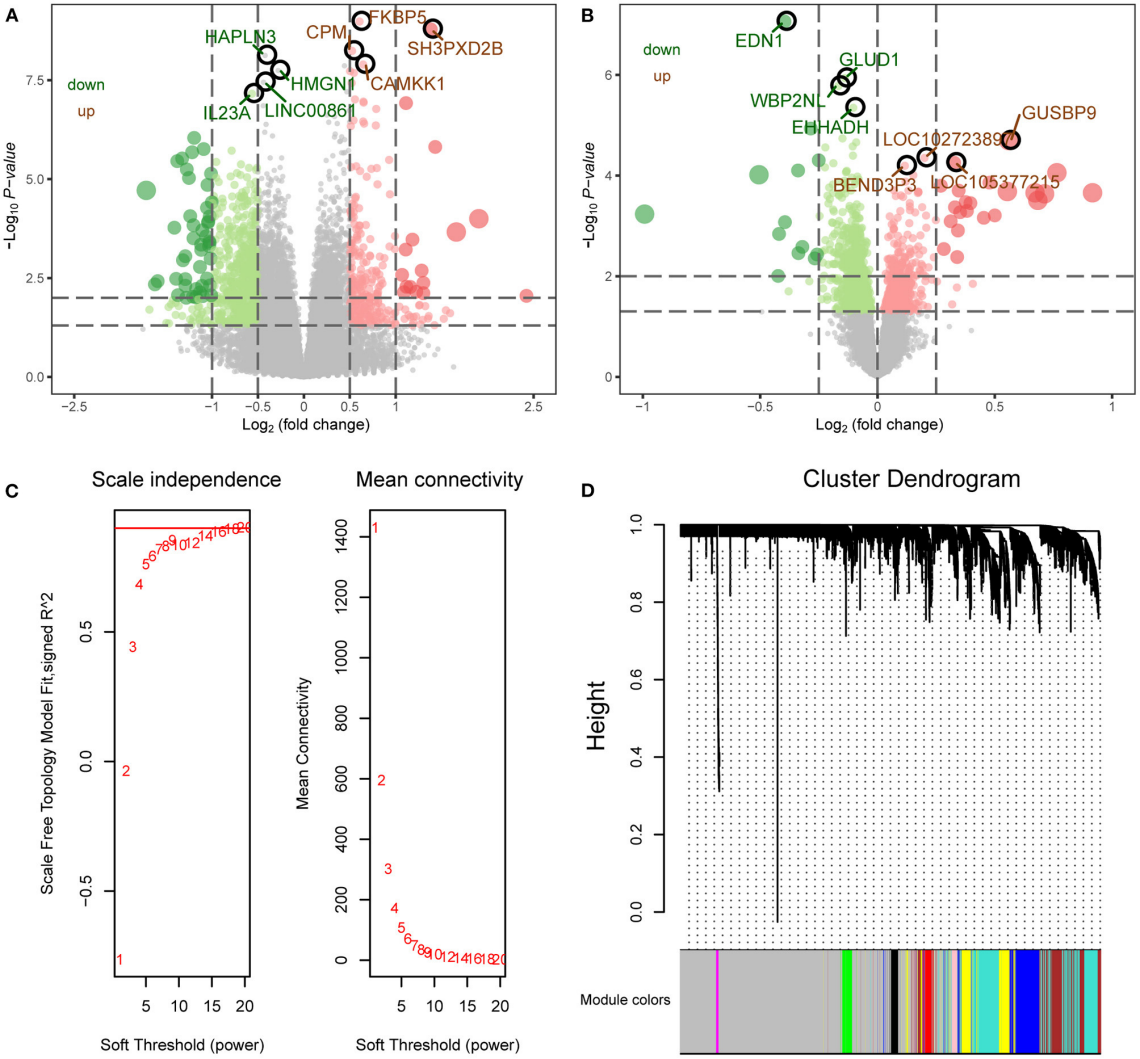
WGCNA network of differentially expressed genes. **(A)** The differentially expressed genes between TBI and control in GSE89866 data. Red nodes were significantly up-regulated genes, and green nodes were significantly down-regulated genes. **(B)** The differentially expressed genes between TBI and control in GSE104687 data. Red nodes were significantly up-regulated genes and green nodes were significantly down-regulated genes. **(C)** Scale free fitting index analysis and average connectivity analysis of different soft threshold (β). The left image shows the scale-free fit index (*y*-axis) as a function of the soft-thresholding power (*x*-axis). The right image shows the average connectivity (degree, *y*-axis) as a function of the soft-thresholding power (*x*-axis). **(D)** The coexpression modules were constructed by the amalgamation of differentially expressed genes in two groups. Different colors represent different modules.

The flowchart of this study is shown in Figure 1. To identify the gene expression characteristics of TBI, we compared the differences between TBI and control in the two datasets. We found 4817 DEGs in GSE89866 (Figure 2A; Supplementary Table 1), including 2,239 up-regulated DEGs and 2,578 down-regulated DEGs. There were 1,329 DEGs in GSE104687, including 518 DEGs up-regulated and 811 DEGs down-regulated (Figure 2B; Supplementary Table 2). The combined genes of two groups of DEGs were subjected to WGCNA, and the soft-thresholding power was selected as 16 (Figure 2C). We identified a total of nine modules (Figure 2D).

### Function of Important Modules

**Figure 3:**
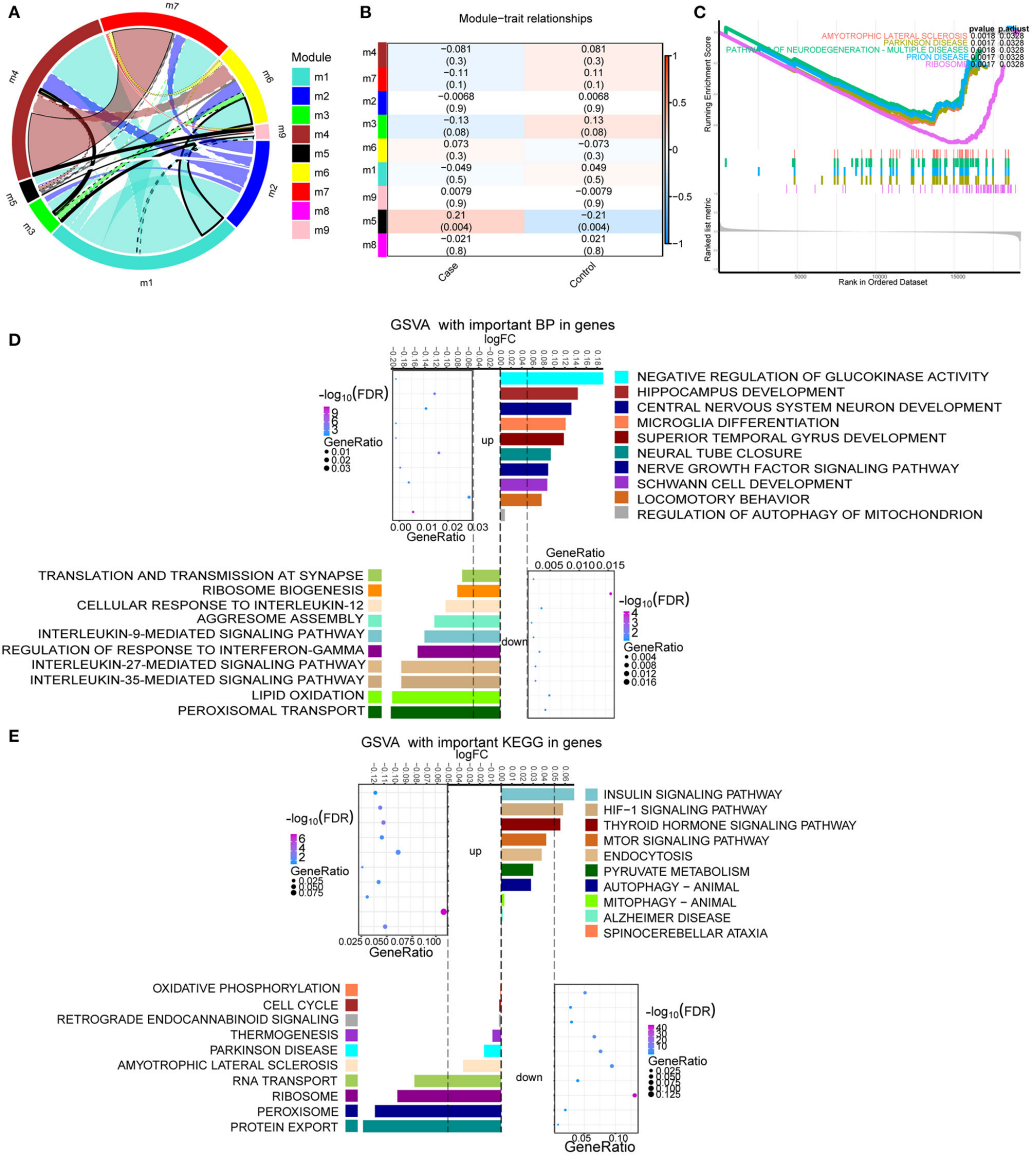
Enrichment analysis of module genes with the most crosstalk. **(A)** Crosstalk between module genes. **(B)** The correlation between module and clinical trait. Red node represents up-regulation, and blue node represents down-regulation. **(C)** The same KEGG signaling pathway in GSEA results as enrichment results. Different colors represent different signaling pathways. **(D)** The up- or down-regulation of major biological processes in important modules calculated by GSVA. **(E)** The up- or down-regulation of the major KEGG pathway in important modules calculated by GSVA.

By analyzing the crosstalk between different module genes, we found that the genes in MEturquoise (module 1), MEbrown (module 4), and MEred (module 7) had the most crosstalk with other module genes, respectively (Figure 3A). Therefore, these modules were identified as important modules. The correlation analysis results showed a negative correlation between these modules and TBI (Figure 3B). There were four identical KEGG pathways in the results of GSEA and enrichment [amyotrophic lateral sclerosis, Parkinson's disease (PD), pathways of neurodegeneration-multiple disease, and ribosome] (Figure 3C). In the enrichment results of GO, we found a large number of TBI-related biological processes (BP), and calculated the up- or down-regulation of terms by GSVA (Figure 3D). Among them, central nervous system neuron development, hippocampus development, nerve growth factor signaling pathway, and microglia differentiation significantly up-regulated enrichment. Interleukin-9,−12,−27, and−35-mediated signaling pathway and regulation of response to interferon-γ were significantly down-regulated. In addition, the main KEGG enrichment results were also evaluated by GSVA (Figure 3E). Spinocerebellar ataxia, pyruvate metabolism, mTOR signaling pathway, mitophagy animal, and HIF-1 signaling pathway were significantly up-regulated. Ribosome, cell cycle, oxidative phosphorylation, and PD were significantly down-regulated.

### Identification of Key Module Genes

**Figure 4:**
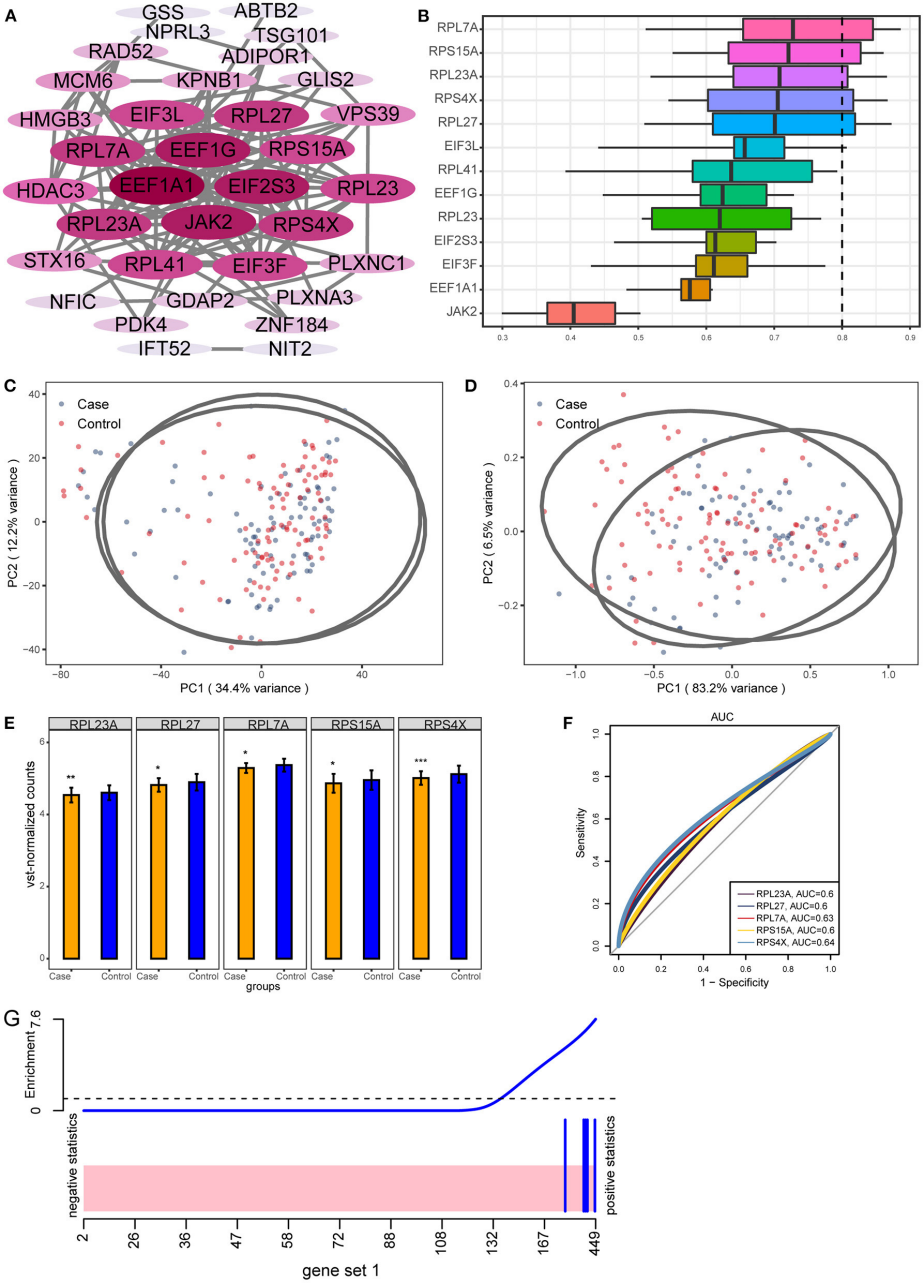
Key genes with potential diagnostic role for TBI. **(A)** The PPI network of 50 genes with larger AUC values. The darker the color, the more connected the gene is in the network. **(B)** GO functional similarity of the top 10 genes with the greatest connectivity. **(C)** Primary component analysis of TBI and control in GSE104687. **(D)** Distinguishing TBI and control samples in GSE104687 using hub genes expression. **(E)** The differential expression of hub genes between TBI and control in GSE104687. *P < 0.05, **P < 0.01, ***P < 0.001. **(F)** The AUC values of hub genes. **(G)** Gene set analysis barcode plot. The differential gene expression in hub genes is shown as a shaded rectangle; genes up-regulated are shaded pink.

By calculating the area under curve (AUC) values of important module genes in GSE104687, we screened the top 50 genes. Further, we performed PPI network analysis for 50 genes (Figure 4A). Genes with connectivity >10 in the PPI network were subjected to perform functional similarity analysis (Figure 4B). We found that RPL27, RPS4X, RPL23A, RPS15A, and RPL7A had high functional similarities (>0.8) and were then identified as hub genes. By performing principal component analysis (PCA) on TBI and control samples in GSE104687 data, we found that the sample distances between the two groups were close (Figure 4C). When using hub genes for PCA, the discrimination between TBI and control can be improved (Figure 4D). Compared with control, hub genes were significantly down-regulated in TBI (Figure 4E). This down-regulation difference was also validated in GSE89866 (Supplementary Figure 1). In addition, the AUC values of hub genes were all >0.6, which may have a diagnostic role for TBI (Figure 4F). Using five hub genes as a gene set, they also down-regulated expression in TBI (Figure 4G).

### Immune Cell Infiltration in TBI

**Figure 5:**
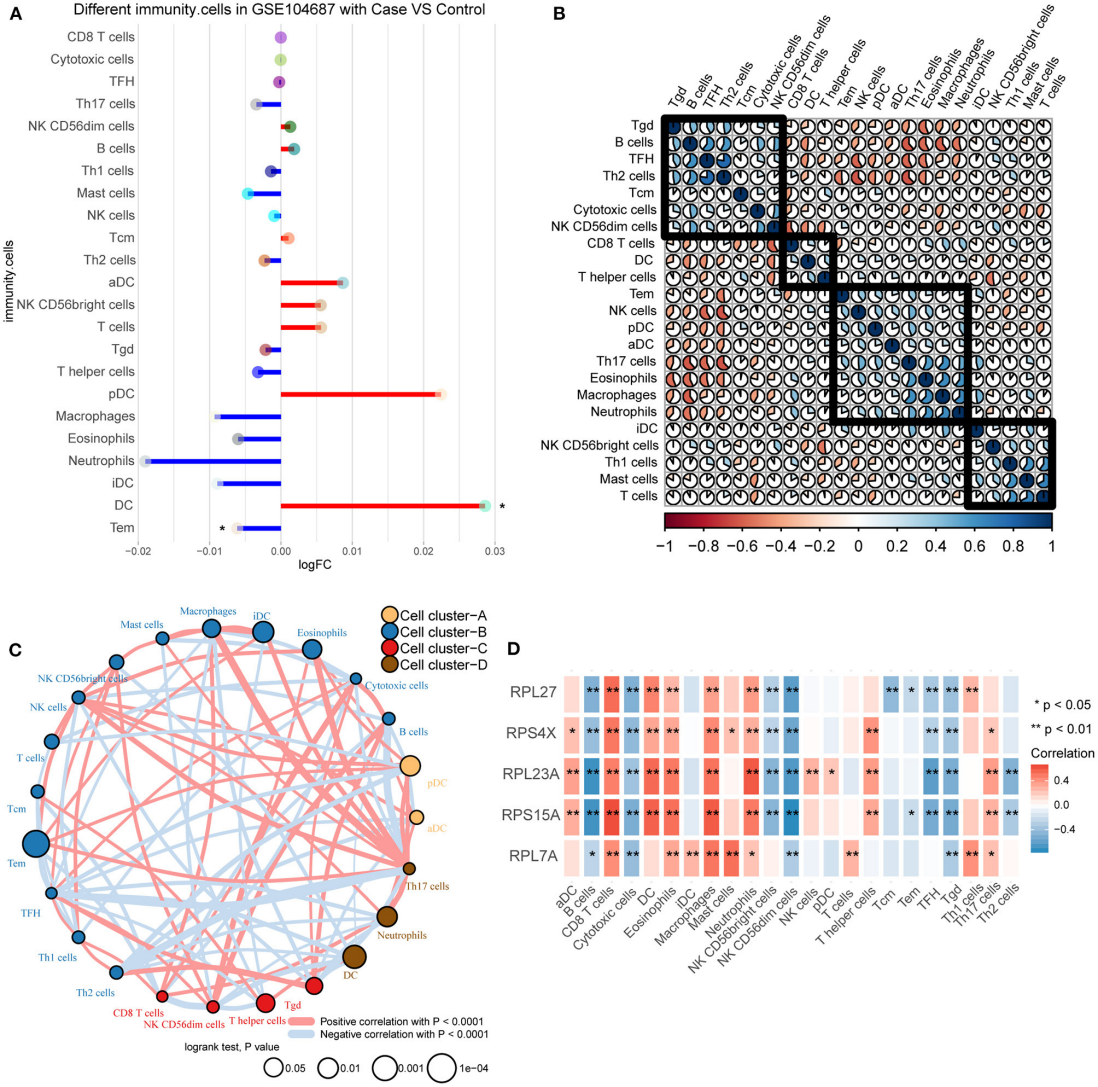
Differences in immune infiltration between TBI and control. **(A)** Differences of immune cell infiltration between TBI and control in GSE104687. Red line represents up-regulation, and blue line represents down-regulation. **(B)** The correlation between immune cells. **(C)** The immune cells were clustered into four groups. **(D)** The correlation between immune cells and hub genes. Red is positive correlation, and blue is negative correlation. *P < 0.05, **P < 0.01.

By comparing the immune cell infiltration between TBI and control, we found that DC was significantly up-regulated and Tem was significantly down-regulated (Figure 5A). By calculating the correlation between immune cells, we found a positive or negative interaction relationship between differentially infiltrated immune cells (Figure 5B). Immune cells were further clustered into four categories by cluster analysis (Figure 5C). The results of correlation analysis showed that the correlation between these immune cells and hub genes was similar (Figure 5D). Among them, RPL23A and RPS15A have strong correlation with immune cells.

## Discussion

Since the process of numerous pathophysiological events occurring after brain injury is extremely complex, it is a great challenge to find the mechanism of molecular changes of TBI. Our study identifies potential therapeutic targets and corresponding molecular mechanisms by exploring the gene expression characteristics of TBI. The novelty of this work lies in the fact that we not only identified potential key genes using multiple sets of data but also associated with immune cells to provide more possibilities for the treatment of TBI.

By coexpression analysis for DEGs, we identified gene sets (modules) with coexpression patterns. Each module may characterize different molecular mechanisms of action (Castranio et al., 2018). Among them, the crosstalk between modules 1, 4, and 7 was the most obvious. There was a negative correlation between these modules and TBI. These module genes were positively associated with the nervous system development and negatively correlated with inflammatory response. The genes of these modules may have a protective effect on the damaged brain. Explosive and non-explosive-induced TBI usually causes white matter and gray matter damage, which may lead to neuronal and/or glial cell damage (Cernak and Noble-Haeusslein, 2010). Apoptosis of neurons and oligodendrocytes is a hallmark of secondary brain injury (Grady et al., 2003). TBI has been reported to cause loss of cortical and hippocampal neurons and alterations in neurotransmitter expression and function (Bondi et al., 2015). Recently, immune inflammation has received extensive attention in the process of TBI. Sterile immune responses can be generated within minutes after TBI, including local signals from neurons, glial cells, and peripheral immune cells, which induce an inflammatory cascade (Corps et al., 2015). After TBI, peripheral blood leukocytes increased significantly, releasing complement factors and proinflammatory cytokines (Dalle Lucca et al., 2012). The sustained up-regulation of various cytokines is associated with changes in blood–brain barrier permeability, edema formation, and neurological deficits (Royes and Gomez-Pinilla, 2019). Interferon-γ can regulate neuronal networks and is associated with more severe disability in the acute phase after brain injury (Kramer et al., 2019).

On the other hand, the mammalian target of rapamycin (mTOR) pathway plays an important role in a variety of physiological functions of the nervous system, such as nerve cell growth, survival, development of dendritic cells during differentiation, and synaptic plasticity (Don et al., 2012). Some studies have shown that mTOR inhibition prevents neuronal injury and death after TBI, while others have shown that increased mTOR signaling after injury promotes cell regeneration and functional recovery (Rana et al., 2019). The occurrence of mitochondrial autophagy has been reported after TBI and is a powerful target (Chu et al., 2013). Oxidative stress response may also be a potential therapeutic target for TBI (Kochanek et al., 2015). Hypoxia may be the driving force of angiogenesis after moderate and severe TBI (Salehi et al., 2017). Experimental TBI animal models revealed the up-regulation of HIF-1 in the injured brain (Park et al., 2009). In fact, TBI is considered as an environmental risk factor for many neurodegenerative diseases, such as PD (Jafari et al., 2013).

The hub genes we identified were all ribosomal subunits. Ribosomal defects can lead to elevated ROS and activation of the TP53 pathway, which have important links with TBI (Sulima et al., 2019). The differential expression of RPL27 and RPS15A was validated in TBI mice (Harper et al., 2020). Recent studies have shown that RPL7 and RPL23A are differentially expressed in senile dementia and may be potential biomarkers (Shigemizu et al., 2020). RPS4X interacts with exogenous lactate dehydrogenase A (LDHA) in the central nervous system and is critical for the proliferation of vascular endothelial cells (Lin et al., 2018). The differential down-regulated expression of hub genes was verified in the two datasets. Importantly, although not completely separated, the hub genes we identified allow more distinct discrimination between samples and will benefit for the diagnosis of TBI in our analysis results.

TBI can induce cell-mediated immune response; however, the role of immunity after TBI is not fully understood. The post-traumatic immune response is rapid and attracts immune cells into the injury site mainly through the early release of cytokines and chemokines (Mayer et al., 2019). Although this inflammatory cascade is necessary for tissue repair and immune defense at the site of injury, an excessive inflammatory response may lead to an inflammatory state (Hildebrand et al., 2006). Dendritic cells can release chemokines and cytokines and promote intercellular and distal signaling at the site of injury through the circulatory system, thereby amplifying the immune response (Jassam et al., 2017). Unlike our analysis, effector memory T cells (Tem) populations were up-regulated in TBI (Ritzel et al., 2018). Immune responses in TBI are now considered both damaging and beneficial (Jarrahi et al., 2020). If regulated, the traumatized brain can benefit from inflammation.

This study also had some limitations. Firstly, the data sample size of our analysis was small, and we needed to expand the sample size for validation analysis. Secondly, our main analysis results lacked the validation of molecular experiments. Finally, whether the identified potential markers have clinical diagnostic role remains to be further studied and verified.

## Conclusion

TBI remains a complex, multisystem pathology with potential for a wide range of short- and long-term harmful outcomes. We identified possible biomarkers and therapeutic targets using gene expression features of TBI in public databases. The RPL27, RPS4X, RPL23A, RPS15A, and RPL7A we identified may have differential effects on TBI. In addition, neurological and immunoinflammatory responses are the main dysregulated mechanisms of TBI. New understanding of these genes will lead to new therapeutic targets with the hope of improving outcomes for TBI patients.

## Data Availability Statement

The original contributions presented in the study are included in the article/Supplementary Material, further inquiries can be directed to the corresponding author.

## Author Contributions

YX conceived and designed the project. YX and YM contributed to the design of the study, writing the protocol, data preparation, analysis, and interpretation. YM, YL, XR, XL, and JZ drafted the manuscript. All authors have read and approved the submitted version.

## Conflict of Interest

The authors declare that the research was conducted in the absence of any commercial or financial relationships that could be construed as a potential conflict of interest.

## Supplementary Material

Supplementary Figure 1 The differential expression of hub genes between TBI and control in GSE89866. ^*^*P* < 0.05, ^**^*P* < 0.01, ^***^*P* < 0.001.

Supplementary Table 1 The differentially expressed genes in GSE89866.

Supplementary Table 2 The differentially expressed genes in GSE104687.
