# Supplementary figures and images for: Gene Expression Signature of Traumatic Brain Injury
Source: Front Genet. 2021 Mar 30;12:646436. doi: 10.3389/fgene.2021.646436 (PMC8042258; doi:10.3389/fgene.2021.646436)

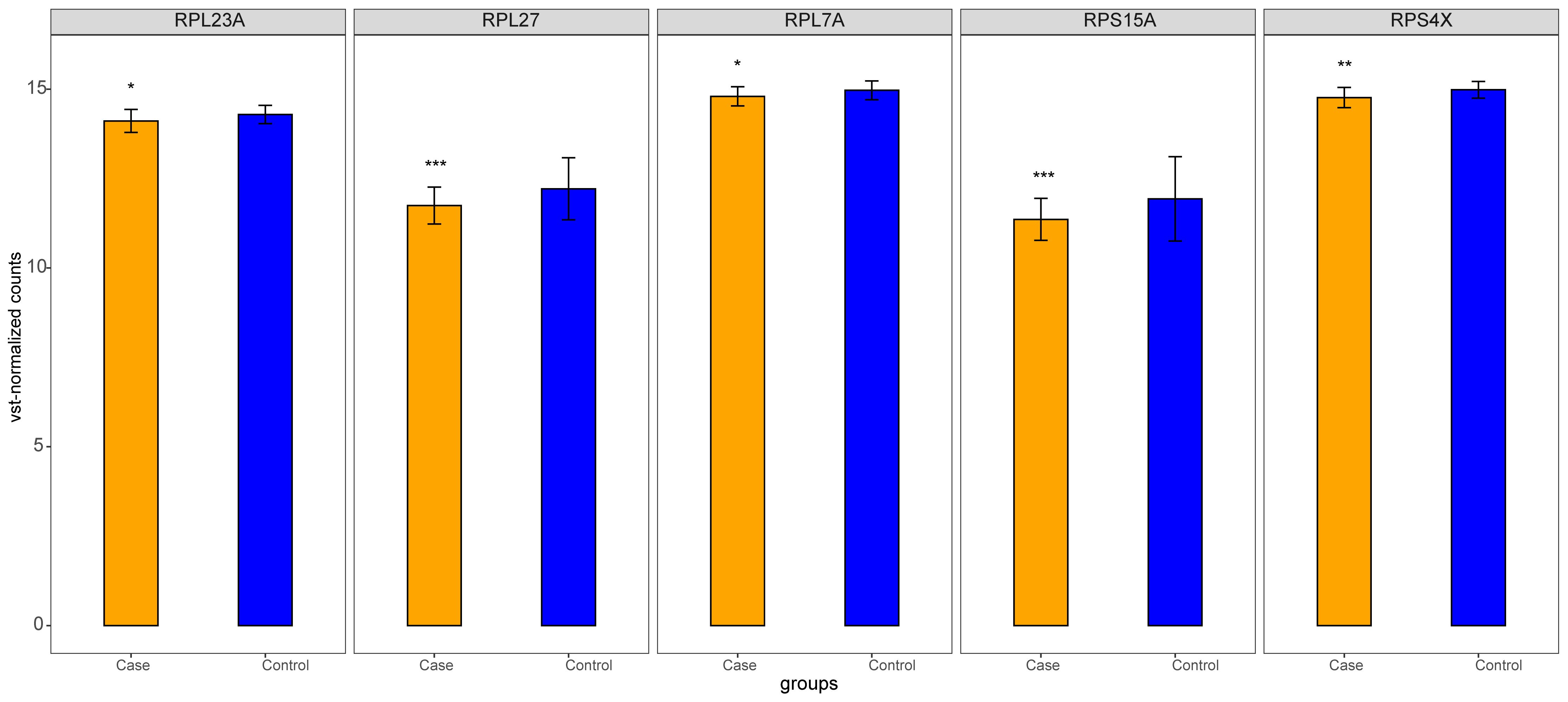

Supplement: Supplementary Figure 1 — The differential expression of hub genes between TBI and control in GSE89866. *P < 0.05, **P < 0.01, ***P < 0.001. [file Image_1.TIF]
